# Burden of non-communicable diseases among the Orang Asli community and patient satisfaction on non-communicable diseases management at public health facilities

**DOI:** 10.1186/1471-2458-12-S2-A28

**Published:** 2012-11-27

**Authors:** Netty Darwina, Sharifa Ezat Wan Puteh

**Affiliations:** 1 Department of Community Health, Universiti Kebangsaan Malaysia Medical Centre, Jalan Yaacob Latiff, 56000 Kuala Lumpur, Malaysia

## Background

Health status of aboriginal or indigenous as general is poor compared to the general population all over the world. The increasing trend of non-communicable diseases (NCD)among Orang Asli community became alarming to the health status of this marginalized group. The crude prevalence rate of diabetic and impaired glucose tolerance (IGT) to be 1.3 and 10.7%, respectively among the Orang Asli population living rural area was significantly higher compared to those living in the jungle (0.0 and 3.3% respectively) and the prevalence of hypertension among Orang Asli community was 30.8% which is lower than the National Health and Morbidity Survey. Patient satisfaction towards health care among Orang Asli or aborigines will further determine and discuss the degree of patient satisfaction about healthcare services for this marginalized group especially regarding NCD. Thus this study is conducted to measure burden of NCD among Orang Asli community and patient satisfaction on NCD management at public health facilities. This is essential to ensure the equality and the equity of health services available for this marginalized group.

## Materials and methods

A cross sectional study among the Orang Asli population with chronic diseases will be conducted in September 2012 at selected Orang Asli villages in Selangor, Pahang and Perak. 650 of the respondents will be selected by using Multistage Sampling Technique.

## Results

Burden of NCD among Orang Asli community and patient satisfaction on NCD management at public health facilities will be measured by using validated patient satisfaction questionnaire with added component of NCD burden.

## Conclusions

This study outcome will serve as information for the policy makers about the burden and patient satisfaction on NCD management among Orang Asli population towards improvement of health programme planning.

